# A meta-analysis of randomized controlled trials evaluating the effectiveness of fecal microbiota transplantation for patients with irritable bowel syndrome

**DOI:** 10.1186/s12876-024-03311-x

**Published:** 2024-07-05

**Authors:** Yu Wang, Yongmei Hu, Ping Shi

**Affiliations:** Yuncheng Central Hospital affiliated of Shanxi Medical University, Shanxi, 044000 China

**Keywords:** Gastrointestinal diseases, Fecal microbiota transplantation, Irritable bowel syndrome, Randomized controlled trials meta-analysis

## Abstract

**Objective:**

Multiple randomized controlled trials (RCTs) have investigated the efficacy of fecal microbiota transplantation (FMT) for irritable bowel syndrome (IBS), but have yielded inconsistent results. We updated the short-term and long-term efficacy of FMT in treating IBS, and performed a first-of-its-kind exploration of the relationship between gut microbiota and emotions.

**Methods:**

We conducted a comprehensive search of PubMed, Embase, Web of Science, and the Cochrane Library using various search strategies to identify all eligible studies. The inclusion criteria for data extraction were randomized controlled trials (RCTs) that investigated the efficacy of fecal microbiota transplantation (FMT) compared to placebo in adult patients (≥ 18 years old) with irritable bowel syndrome (IBS). A meta-analysis was then performed to assess the summary relative risk (RR) and corresponding 95% confidence intervals (CIs).

**Results:**

Out of 3,065 potentially relevant records, a total of 10 randomized controlled trials (RCTs) involving 573 subjects met the eligibility criteria for inclusion in the meta-analysis. The meta-analyses revealed no significant differences in short-term (12 weeks) (RR 0.20, 95% CI -0.04 to 0.44), long-term (52 weeks) global improvement (RR 1.38, 95% CI 0.87 to 2.21), besides short-term (12 weeks) (SMD − 48.16, 95% CI -102.13 to 5.81, I^2^ = 90%) and long-term (24 weeks) (SMD 2.16, 95% CI -60.52 to 64.83, I^2^ = 68%) IBS-SSS. There was statistically significant difference in short-term improvement of IBS-QoL (SMD 10.11, 95% CI 0.71 to 19.51, I^2^ = 82%), although there was a high risk of bias. In terms of long-term improvement (24 weeks and 54 weeks), there were no significant differences between the FMT and placebo groups (SMD 7.56, 95% CI 1.60 to 13.52, I^2^ = 0%; SMD 6.62, 95% CI -0.85 to 14.08, I^2^ = 0%). Sensitivity analysis indicated that there were visible significant effects observed when the criteria were based on Rome IV criteria (RR 16.48, 95% CI 7.22 to 37.62) and Gastroscopy (RR 3.25, 95%CI 2.37 to 4.47), Colonoscopy (RR 1.42, 95% CI 0.98 to 2.05). when using mixed stool FMT based on data from two RCTs, no significant difference was observed (RR 0.94, 95% CI 0.66 to -1.34). The remission of depression exhibited no significant difference between the FMT and placebo groups at the 12-week mark (SMD − 0.26, 95% CI -3.09 to 2.58), and at 24 weeks (SMD − 2.26, 95% CI -12.96 to 8.45). Furthermore, major adverse events associated with FMT were transient and self-limiting.

**Discussion:**

Based on the available randomized controlled trials (RCTs), the current evidence does not support the efficacy of FMT in improving global IBS symptoms in the long term. The differential results observed in subgroup analyses raise questions about the accurate identification of suitable populations for FMT. Further investigation is needed to better understand the reasons behind these inconsistent findings and to determine the true potential of FMT as a treatment for IBS.

**Supplementary Information:**

The online version contains supplementary material available at 10.1186/s12876-024-03311-x.

## Introduction

For Irritable bowel syndrome (IBS) is characterized by abdominal pain, bloating, and discomfort, often accompanied by changes in bowel habits such as frequency and consistency [1]. A persistent disorder marked by varying symptom severity, frequently overlapping with other functional disorders and psychiatric conditions [2]. IBS has a global prevalence ranging from 5.8 to 17.5%, as estimated from pooled regional data [3]. IBS patients often experience comorbid depression and a decreased quality of life (QOL). While not directly increasing mortality, the condition significantly affects health-related quality of life (QoL), healthcare costs, and work productivity [4, 5]. The disorder can be categorized into four subtypes based on the predominant bowel habits: diarrhea-predominant IBS, constipation-predominant IBS, mixed IBS, and unclassified IBS [6].

Previous studies indicate the pathogeneis includes various factors such as genetic factors, visceral hypersensitivity, inflammatory agents, disturbances in gut-brain interaction, or psychosocial stress which is one of its pathogenesis [6, 7]. As research on IBS advances, progress in the understanding of the brain-gut axis has revealed a close relationship between gut microbes and emotions [8–10]. The brain-gut axis, which is the bi-directional, neurohumoral communication system connecting the gut and brain, through interactions involving the autonomic nervous system, the HPA axis, and the microbiome, serves as the primary physiological connection between IBS and depression and anxiety [11]. Even in healthy individuals, stress can impair gut function by causing the autonomic nervous system to produce corticotrophin-releasing factor [12]. In individuals with IBS, the dysregulation of the HPA axis and high activity in the amygdala contribute to a heightened susceptibility to and reduced recovery from stressful events [13–17]. This reduced resilience to stress is associated with the co-existence of depression and IBS [18]. The microbiome regulates gastrointestinal function and plays a crucial role in gut-brain communication [11]. Its composition differs among people with and without depression, as well as among IBS patients with and without psychological comorbidity [19–21]. Some studies suggest probiotics may benefit mood disorders and IBS symptoms. Animal models also show that the microbiome affects brain-gut interaction, as stool transplants from depressed or anxious individuals with IBS into mice cause inflammatory and behavioral changes [22, 23]. Co-occurrence of depression and IBS is estimated to be between 44% and 84% [24], also suggesting a possible connection between these conditions. Liu et al. found that the fecal microbiota profiles in patients with depression were similar to those of IBS-D patients [25]. Genetic factors, visceral hypersensitivity, inflammatory agents, disruptions in gut-brain interaction, or psychosocial stress can all lead to an imbalance in the gut microbiota, known as dysbiosis. This imbalance can cause disruptions in the integrity of the mucosal epithelium and gastrointestinal motility [26, 27].

“On the gut microbiome” for IBS treaments, and explored diverse approaches in its manipulation such as antibiotics, probiotics, encompassing prebiotics, the modifications of dietary [28–30]. Many patients remain symptomatic who were treated as described above, indicating the need for more effective treatments. FMT is an innovative treatment approach designed to rebalance the gut microbiota by transferring fecal microbiota to the patient’s gastrointestinal tract which is from a healthy donor. This transfer can be done through oral capsules, nasojejunal administration, or endoscopic procedures [31]. FMT, with minimal and self-limited adverse effects, has proven effective in treating a range of gastrointestinal disorders, including inflammatory bowel disease, recurrent Clostridium difficile infection, chronic constipation, hepatic encephalopathy, and colorectal cancer [32]. However, the effectiveness of FMT in treating IBS remains a subject of debate.

Recent studys conducted a systematic review and meta-analysis of the published RCTs [33–35], while there is no systematic review analyzing the efficacy of FMT for patients with IBS and comorbid depression. In this review, we aimed to extend the work of El-Salhy et al. [36] by updating the evidence, expanding the outcomes to include the global improvement, IBS-SSS, IBS-QOL and depression.

## Materials and methods

Meta-analyses were conducted following the Preferred Reporting Items for Systematic review and Meta-analyses (PRISMA) guidelines.

### Search strategies and research options

We conducted a comprehensive search using five electronic databases, including PubMed, Embase, the Cochrane Library, and Web of Science, covering the period from inception to December 14, 2023. Additionally, unpublished trials and supplementary data were identified by manually searching Clinicaltrials.gov to ensure no studies were missed. The detailed search strategy is outlined in Supplementary Appendix 1.

We performed a meta-analysis specifically focused on randomized controlled trials that investigated fecal microbiota transplantation in patients diagnosed with IBS using either Rome III or IV criteria. The intervention involved fecal microbiota transplant administered through various routes and dosages, compared to autologous transfer or a control group. The main outcome measure was the alteration in the severity of IBS symptoms assessed through the IBS-SSS scale at various time points. Additionally, we assessed the side effects of the intervention and the safety. Conference abstracts, single-arm trials, and case reports were excluded from our analysis. Our PICO criteria included the following:

Populations: Individuals with IBS.

Interventions: Fecal microbiota transplantation (FMT) with various administration routes and dosages.

Comparisons: Autologous transfer or control group.

Outcomes: Evaluation of alterations in IBS symptom severity and disease control, encompassing an examination of safety and potential side effects of the intervention.

### Outcome assessment

Our study’s primary focus was to assess the effectiveness of Fecal Microbiota Transplantation (FMT) in comparison to a placebo, specifically gauging the response to therapy through the global improvement in IBS symptoms. The global improvement was categorized as a binary outcome, identified through a predetermined threshold distinguishing between response and non-response on either Gastrointestinal Symptom Rating Scale for IBS (GSRS-IBS) total score or the IBS Severity Scoring System instrument (IBS-SSS) score. The secondary outcomes of this study were to assess the improvement in the IBS Severity Scoring System (IBS-SSS) score (i.e., reduction of ≥ 75 points), the increase in quality of life (QoL) scores on IBS-QoL, the occurrence of adverse events (AEs), and depression measured by the Hospital Anxiety and Depression Scale. We collected data for two time frames: “short-term” outcomes were defined as 8 to 12 weeks, while “long-term” outcomes spanned 6 to 12 months [37, 38]. We gathered data from each study to identify the longer duration within the short-term (8 to 12 weeks), and the long-term (6 to 12 months) periods. For example, if a study reported outcomes at both 8 and 12 weeks, our study prioritized the 12-week data for our meta-analyses.

### Data extraction

Data extraction was conducted independently by two reviewers using Microsoft Excel spreadsheet. The following data points were collected for each study: (1) study characteristics including authors, country, study type; (2) Patient characteristics included number of patients, sex, mean age, diagnostic criteria, IBS subtypes and so on; (3) Placebo preparation; (4) Stool donor details encompassed stool preparation methods and the number of donors; (5) The specific FMT methods, such as preparation, route, frequency, and duration, were recorded; (6) The primary and the secondary outcomes; (7) Additional data covered the duration of follow-up post FMT, country of origin, FMT-related adverse events, number of centers, number of FMT treatments, FMT modality, therapy duration, criteria defining IBS, total reported adverse events, primary outcome measures for symptom improvement, and follow-up duration. Intention-to-treat analyses were conducted, assuming drop-outs as treatment failures, and any unclear information was clarified with the original investigators.

### Assessment of quality and risk of bias

At the individual study level, two investigators independently evaluated bias risk using the Cochrane risk of bias tool. Disagreements were resolved through discussion. The methodology, including the generation of the randomization schedule, blinding implementation for participants, concealment of treatment allocation, outcome assessment, and personnel, as well as evidence of incomplete outcomes data and selective reporting of outcomes, was systematically documented.

### Data synthesis and statistical analysis

Relative risks (RRs) with corresponding 95% confidence intervals (CIs) were pooled to evaluate the persistence of symptomatic outcomes after fecal microbiota transplantation (FMT) compared to placebo. RRs were employed to assess adverse events (AEs), whereby statistical significance was considered achieved when the 95% CI did not cross 1. Additionally, a mean difference in the quality of life related to irritable bowel syndrome (IBS-QoL) between FMT and placebo was computed, and a random-effect model was employed for data pooling. Subgroup analyses were performed, considering study characteristics such as the risk of bias, FMT administration routes (capsules, nasogastric tube, gastroscopy, or colonoscopy), the types of feces used (frozen or fresh), and donor quality (well, relatively well, or unclearly defined donors). Sensitivity analyses included iteratively conducting the meta-analysis, excluding one study at a time, to evaluate the statistical robustness of the primary outcome. Due to the limited number of identified studies (fewer than 10), we exercised caution in utilizing Egger’s regression asymmetry test and funnel plots for assessing publication bias. The meta-analysis was conducted using RevMan 5.4, a tool provided by The Cochrane Collaboration, The Nordic Cochrane Centre, Copenhagen, Denmark.

Our planned approach for assessing publication biases involved employing Egger’s test in conjunction with funnel plots, specifically in cases where the number of included studies exceeded 10. Furthermore, heterogeneity was evaluated using the I^2^ statistic [39]. To address studies with multiple intervention groups (e.g., varying dosages utilized for the intervention group) within a single study, we followed the recommendation outlined in the Cochrane Handbook to combine these groups and create a single pair-wise comparison. Review Manager (Version 5.4, RevMan for Windows, the Nordic Cochrane Centre, Copenhagen, Denmark) was employed for data analyses.

## Results

**Fig. 1:**
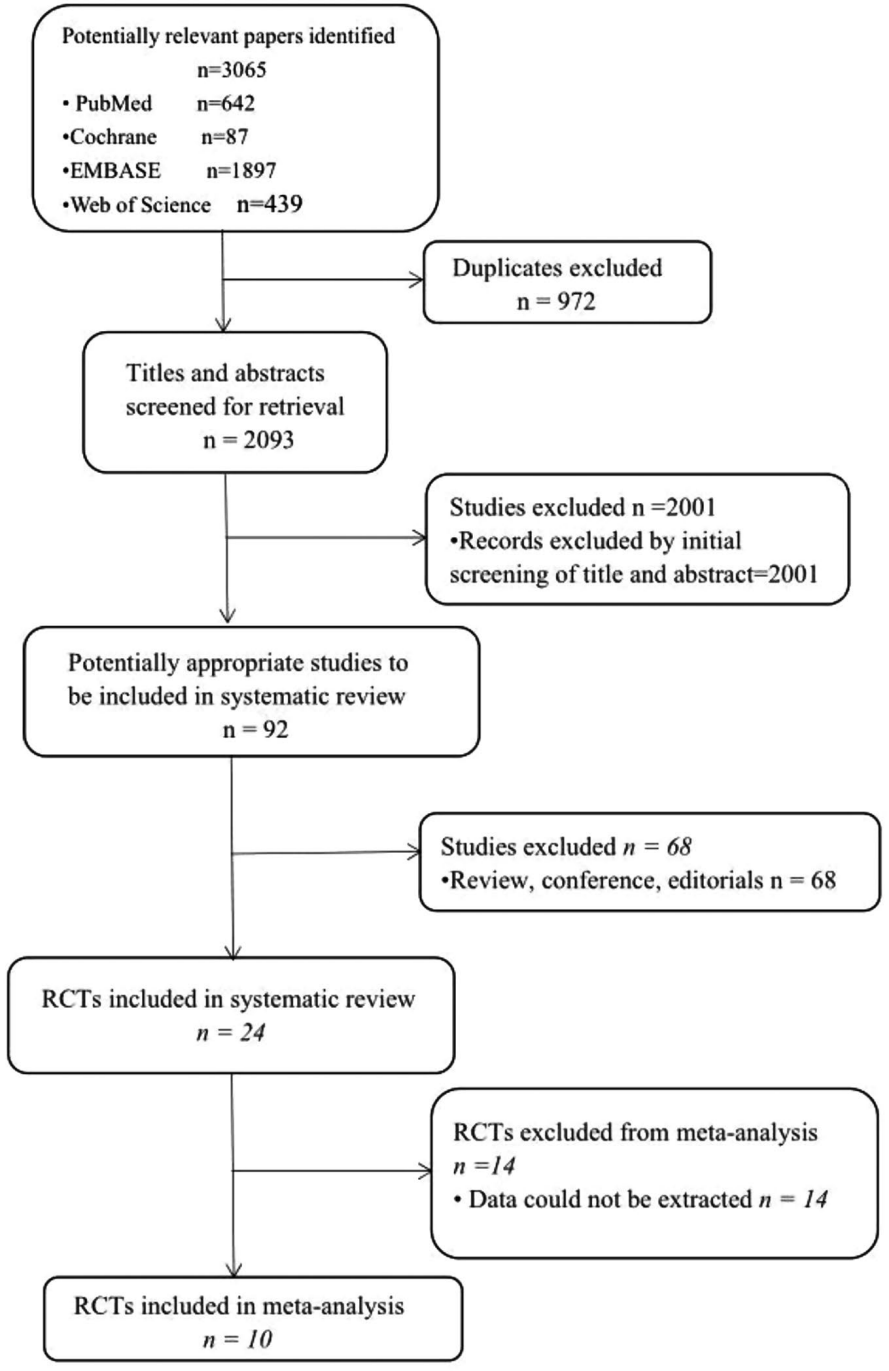
Flowchart of study selection strategy in the systematic review and meta-analysis. RCTs, randomized controlled trials

The search strategy employed yielded a total of 3065 citations. Subsequent to an initial screening of titles and abstracts, 68 citations were subjected to full-text review. Of these, data synthesis included 10 randomized controlled trials (RCTs) containing 573 subjects that met the eligibility criteria for inclusion in the meta-analysis. Five of the studies had one intervention group and one placebo respectively. E-Salhy et al.‘s [36] study included two intervention groups and one placebo group(FMT 30 g and FMT 60 g), Aroniadis et al.’s [6] study conducted a crossover trial, so they are divided into two groups respectively. While El-Salhy et al.‘s 2022 [40] reported the long-term study results of all outcome measures in El-Salhy et al.‘s 2019 [32], additionally, Johnsen et al. [41] reported the secondary outcome measures of Johnsen et al. 2018 [42] so they were combined into one study respectively. As a result, a total of 10 RCTs were included in the analysis. Figure 1 displays the general information of the RCTs included in the analysis. The seven randomized controlled trials (RCTs) were all presented as full-text articles. The characteristics of the included RCTs are detailed in Table 1.

**Table 1:** Characteristics of included RCTs

Study	Country	Diagnostic criteria	Number of center	IBS subtypes		Donors	FMT route and location cleansing (upper/lower GI tract)		
Johnsen et al. [42]	Norway	Rome III	1	44 (53%) IBS-D; 39 (47%)IBS-M		Two donors, mixed	Colonoscopy, Lower		
Johnsen et al. [41]	Norway	Rome III	1	44 (53%) IBS-D; 39 (47%)IBS-M		Two donors, mixed	Colonoscopy, Lower		
Halkjær et al., [43]	Denmark	Rome III	2	17 (33.3%) IBS-C;15 (29.4%) IBS-D; 19 (37.3%)IBS-M		Four donors, mixed	Oral capsules, Upper		
El-Salhy et al. [36]	Norway	Rome IV	3	63(38.4%)IBS-D; 62(37.8%)IBS-C; 39(23.8%)IBS-M		One donor, not mixed	Gastroscopy, Upper		
El-Salhy et al. [40]	Norway	Rome IV	3	47(37.6%)IBS-D; 46(36.8%)IBS-C; 32(25.6%)IBS-M		One donor, not mixed	Gastroscopy, Upper		
Aroniadis et al. [6]	USA	Rome III	3	100% IBS-D		Four donors, not mixed	Oral capsules, Upper		
Holster et al. [44]	Sweden	Rome III	1	4 (25%) IBS-C; 9 (56.3%) IBS-D; 3 (18.8%) IBS-M		Two donors, not mixed	Colonoscopy, Lower		
Lahtinen et al. [46]	Finland	Rome III,	4	51.0% IBS-D, 6.1% IBS-C, 14.3% IBS-M, 28.6% IBS-U		One donor, not mixed	Colonoscopy, Lower		
Holvoet et al. [45]	Belgium	Rome III,	1	100% IBS-D or IBS-M		Two donors; not mixed	Nasojejunal tube, Upper		
Lin et al. [50]	China	Rome III	1	100% IBS-D		One donor, not mixed	Oral capsules, Upper		
**Study**	**Frequency and duration**	**FMT group**		**Control group**		**Primary outcome**		**Secondary outcome**	**Follow-up**
		**Sample size**	**Intervention**	**Sample size**	**Intervention**				
Johnsen et al. [42]	Single	55	FMT consisting of 50–80 g both fresh and frozen (1:1) donor stool via colonoscopy	28	50–80 g autologous stool via colonoscopy	Reduction in the IBS-SSS total score of ≥ 75 points at 3 months		Reduction in IBS-SSS ≥ 75 points at 12 months	12 months
Johnsen et al. [41]	Single	55	FMT consisting of 50–80 g both fresh and frozen (1:1) donor stool via colonoscopy	28	50–80 g autologous stool via colonoscopy	NA		evaluate the fatigue and quality of life	12 months
Halkjær et al. [43]	Multiple: lasting 12 days	25	25 FMT capsules consisting of 50 g frozen donor stool daily × 12 d, from mixed samples of 4 donors	26	25 placebo capsules daily × 12 d	Decrease in IBS- SSS ≥ 50 points at 3 months		Side effects, change in IBS-QoL microbiota profile	6 months
El-Salhy et al. [36]	Single	54(30 g FMT) 55(60 g FMT)	Single FMT consisting of 30–60 g donor frozen stool to the duodenum via gastroscopy, from one super donor	55	Single autologous stool via gastroscopy	Decrease in IBS- SSS ≥ 50 points at 3 months		The change in the dysbiosis index and IBS- QoL, adverse events, microbiota profile	3 months
El-Salhy et al. [40]	Single	42(30 g FMT) 45(60 g FMT)	Single FMT consisting of 30–60 g donor frozen stool to the duodenum via gastroscopy, from one super donor	38	Single autologous stool via gastroscopy	IBS-SSS total score of ≥ 50 points at 2, 3 years		The change in the dysbiosis index and microbiota profile. adverse events	3 years
Aroniadis et al. [6]	Multiple: lasting 3 days, then received placebo capsules at 12 weeks	25	25 FMT capsules consisting of 28 g frozen donor stool daily × 3 d, from single sample of either of the 4 donors	23	25 placebo capsules daily × 3 d	Difference in the IBS-SSS total score at 3 months		Reduction in the IBS-SSS total score of at least 50 points at 3 months; the assessment of differences in QOL, depression, anxiety, stool consistency and microbiome profiles at 3 months	6 months
Holster et al. [44]	Single	8	Single FMT, consisting of 30 g fresh donor stool via colonoscopy, from single sample of either of the 2 donors	8	Single 30 g autologous stool via colonoscopy	Reduction in the GSRS-IBS total score of ≥ 30%		Change of the IBS-SSS, their general health and quality of life (36-item Short Form Survey (SF-36), IBS-QOL, anxiety and depression status	6 months
Lahtinen et al. [46]	Single	23	Single FMT consisting of 30 g frozen donor stool via colonoscopy, from single donor	26	Single 30 g autologous stool via colonoscopy	Reduction in the IBS-SSS total score of ≥ 50 points at 3 months		Changes in IBS-QOL, gut microbiota, fecal water content, intestinal microbiota composition, and stool dry weight.Adverse events	52weeks
Holvoet et al. [45]	Single	43	Single FMT consisting of donor fresh stool to the duodenum via nasojejunal tube from single sample of either of two donors	19	Single autologous stool via nasojejunal tube	Self-reported improvement of overall IBS symptoms and abdominal bloating at 3 months		Changes in daily assessed IBS symptoms, IBS-QOL, change of IBS-related symptoms scores and fecal microbiota transplantation	3 months
Lin et al. [50]	Capsules 3 times in total, once every other Day, 30 capsules each time	9		9	blank capsules	Bristol stool scale (BSS), IBS symptom severity scale (IBS-SSS), and Irritable Bowel Syndrome Quality of Life (IBS-QOL), fecal microbiota		NA	3 months

**Fig. 2:**
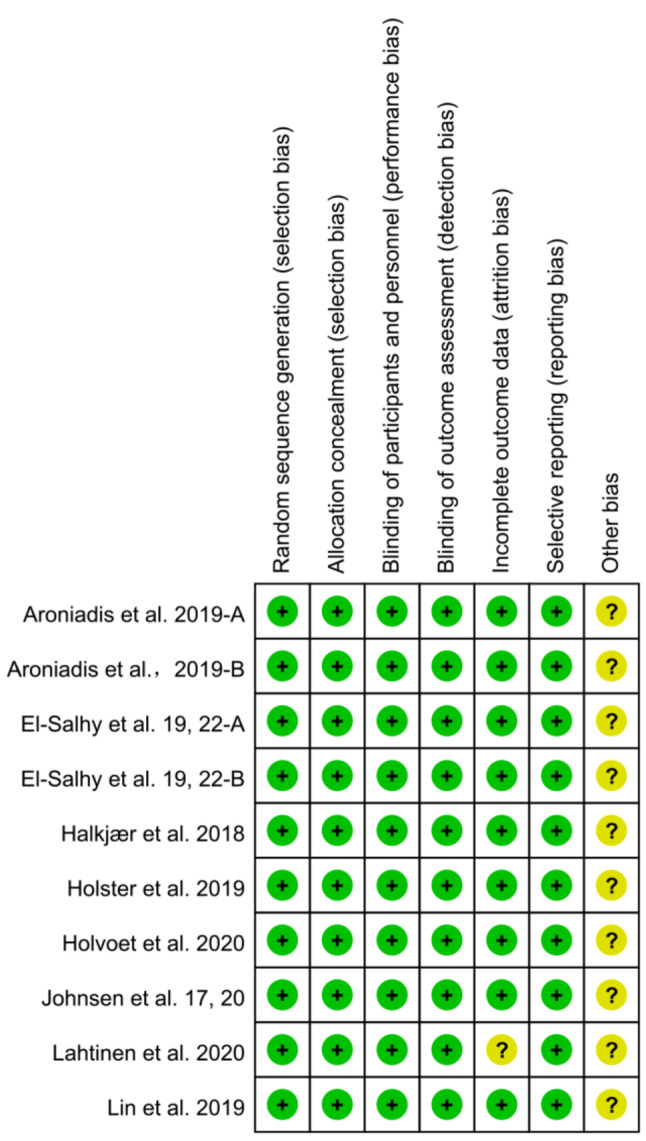
Risk-of-bias assessment of randomized controlled trials using Cochrane risk of bias tool

Figure 2 presents an overview of the risk of bias across studies, assessed using the Cochrane risk-of-bias tool. Nine RCTs were considered to have a low risk of bias, RCTs that reported had an unclear risk of bias due to incomplete outcome data.

### Primary outcome: global improvement

**Fig. 3:**
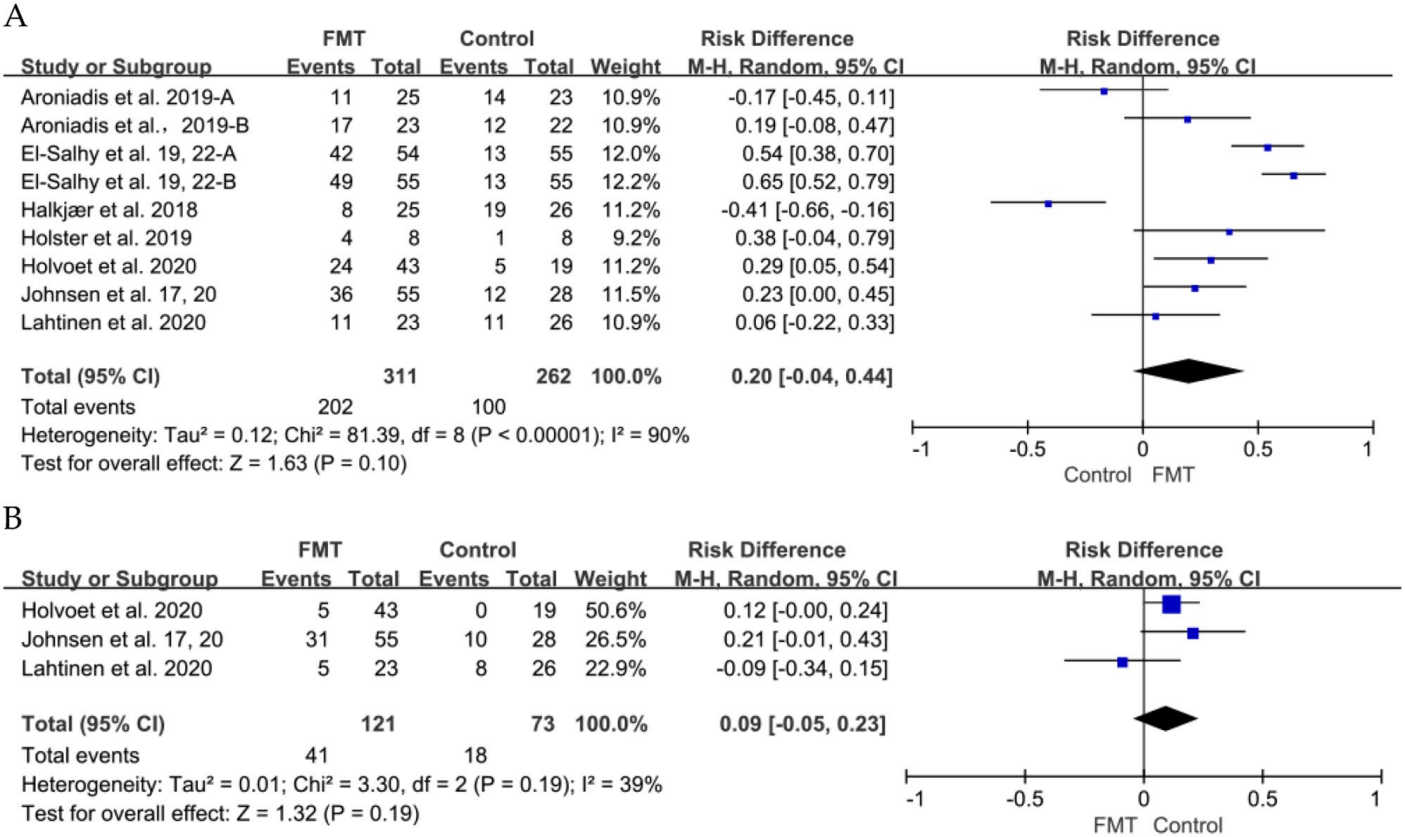
Forest plot of global symptom of IBS between FMT and placebo. (**A**) Short term. (**B**) Long term

There was a total of 9 RCTs [6, 36, 40, 42–46] reported short-term global symptom outcomes, while 3 studies reported long-term outcomes. The primary outcome analysis included 573 patients, with 311 receiving FMT and 262 receiving a placebo. At the 12-week mark, the global improvement in IBS symptoms was 65.0% (202/311) for patients who underwent donor FMT and 38.2% (100/262) for those in the placebo group. No significant improvement was observed at the 12-week mark post FMT compared to the placebo groups (RR = 0.20, 95% CI -0.04 to 0.44, *p* = 0.10). A significant heterogeneity was observed among studies (I^2^ = 90%, *p* < 0.00001) (Fig. 3). Only nine RCTs were included, making it insufficient to assess publication bias.

At 52 weeks, the three studies [42, 45, 46]that reported long term outcomes, 33.9% global improvement in IBS symptoms (41/121) in patients who received donor FMT, 24.7% (18/73) in patients who received a placebo. However, there was no significant difference in global symptom improvement observed between the FMT and placebo groups at 52 weeks (RR 0.09, 95% CI -0.05 to 0.23, I^2^ = 39%) (Fig. 3).

We performed subgroup analyses on the primary outcome, considering various study characteristics. Pooling data from two RCTs, FMT demonstrated superiority over placebo in IBS patients who met the Rome IV criteria (RR 16.48, 95% CI 7.22 to 37.62, I^2^ = 31%). However, when the criteria was based on Rome III, there was no significant effect observed (RR = 1.22, 95% CI 0.45 to 3.32) with high heterogeneity (I^2^ = 74%). The analysis of donor feces composition revealed a statistically significant association between FMT and an increased response rate when using non-mixed donor feces (RR = 1.97, 95% CI 1.04 to 3.71, I^2^ = 82%). However, no significant effect was found when using mixed stool FMT based on data from two RCTs (RR = 0.83, 95% CI 0.24 to 2.83, I^2^ = 90%). Other subgroup analyses also indicated statistically significant differences between FMT and placebo when the data from three RCTs were pooled (Table 2).

**Table 2:** Subgroup analyses comparing FMT with placebo in IBS

	No. of RCTs	RR	95%CI	*P*	I^2^
Route of delivery					
Oral capsules	3	0.77	0.40–1.50	0.45	78%
Gastroscopy	3	3.25	2.37–4.47	< 0.00001	0%
Colonoscopy	3	1.42	0.98–2.05	0.06	0%
**Mixed or single donor sample**					
Mixed	2	0.83	0.24–2.83	0.77	90%
Single	6	1.97	1.04–3.71	0.04	82%
**IBS criteria**					
Rome III	6	1.22	0.45–3.32	0.7	74%
Rome IV	2	16.48	7.22–37.62	< 0.00001	31%
**IBS subtype**					
Non-constipation subtype	4	1.82	0.79–4.18	0.16	55%
All subtype	5	1.77	0.73–4.32	0.21	89%
**FMT dosage**					
Single dose	6	2.24	1.44–3.49	0.0004	66%
Multiple dose	2	0.57	0.35–0.94	0.03	30%

### Secondary outcome: IBS-SSS and IBS-QOL

**Fig. 4:**
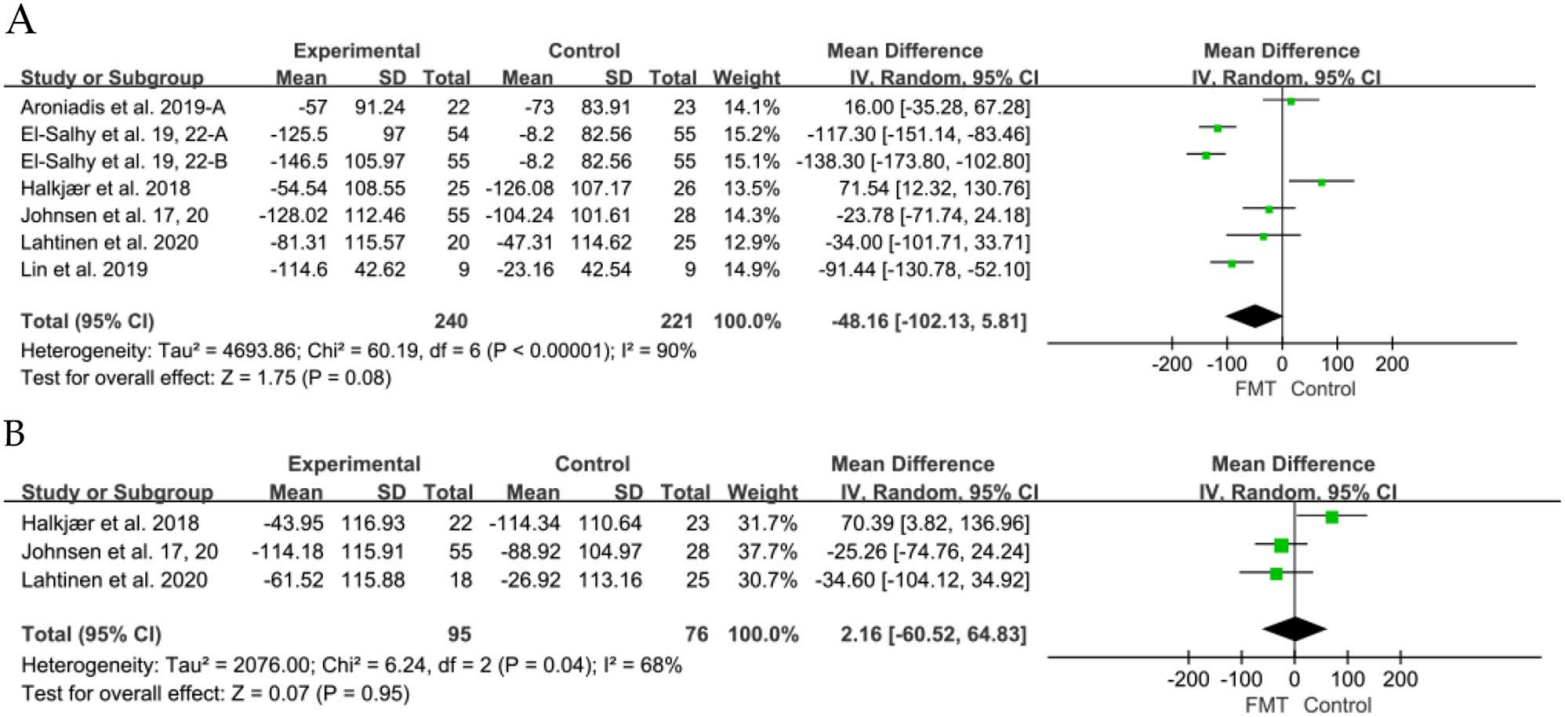
Forest plot of IBS-SSS outcome between FMT and placebo. (**A**) Short term. (**B**) Long term

For secondary outcomes, 7 studies [6, 36, 40, 42, 43, 46, 47] evaluated the short-term improvement of IBS-SSS. No significant improvement was observed at the 12-week mark post FMT compared to the placebo groups (SMD − 48.16, 95% CI -102.13 to 5.81, I^2^ = 90%) (Fig. 4). Three studies investigated the improvement of IBS-SSS between FMT and placebo at 24 weeks [42, 43, 46]. No significant difference in IBS-SSS was observed between patients receiving placebo and those receiving donor FMT (SMD 2.16, 95% CI -60.52 to 64.83, I^2^ = 68%).

**Fig. 5:**
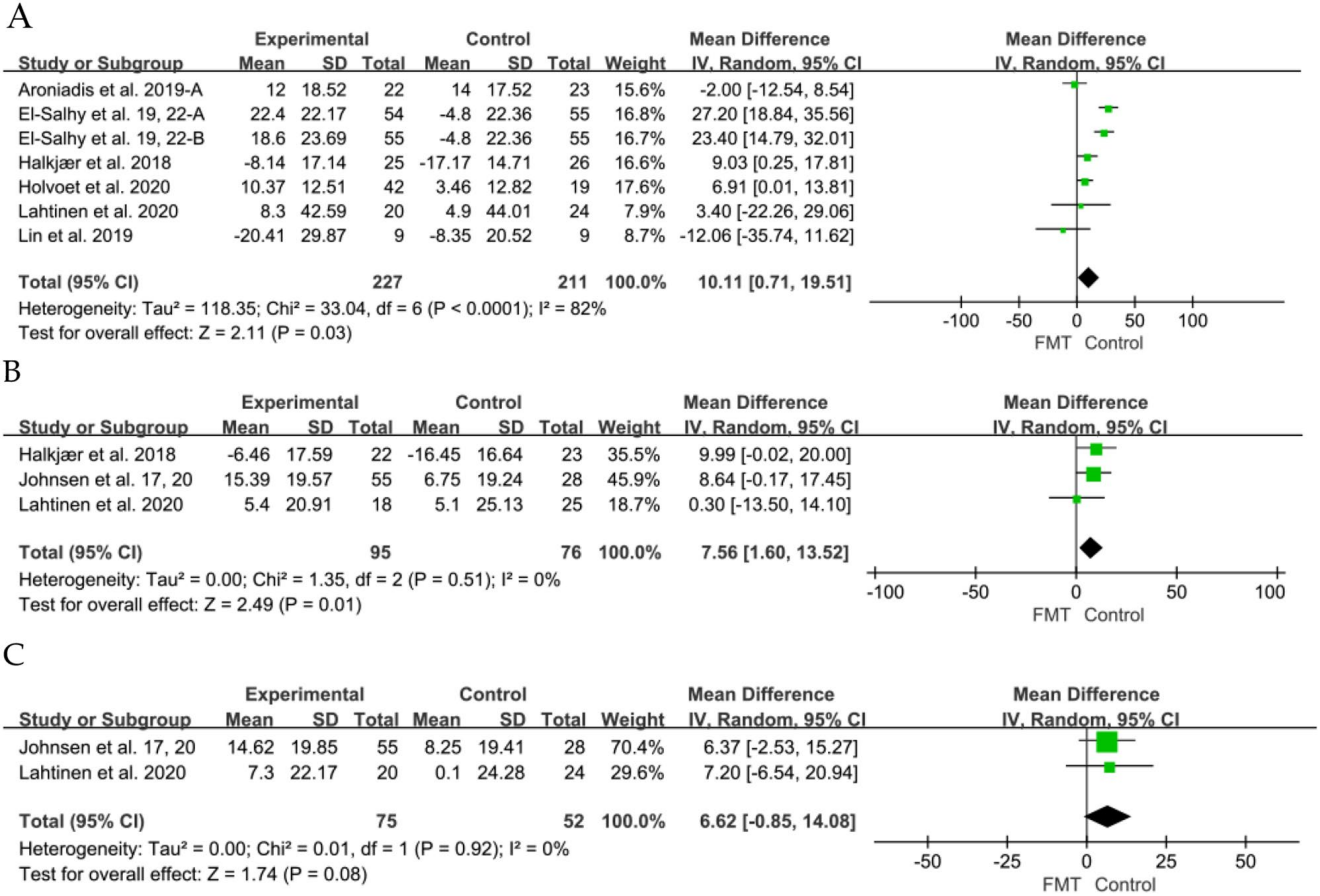
Forest plot of IBS-QoL outcome between FMT and placebo. (**A**) 12 weeks. (**B**) 24weeks. (**C**) 54 weeks

The changes of IBS-QOL were assessed in 7 RCTs [6, 36, 40, 43, 45–47], specifically focusing on the short-term improvement of the IBS-QoL between the placebo and FMT groups at 12 weeks, FMT demonstrated a significant improvement in IBS-QoL compared to the placebo (SMD 10.11, 95% CI 0.71 to 19.51, I^2^ = 82%). Three studies [42, 43, 46] also showed significant difference in long-term IBS-QoL between the FMT and placebo groups at 24 weeks (SMD 7.56, 95% CI 1.60 to 13.52, I^2^ = 0%). No significant difference was observed in IBS-QoL between patients who received donor FMT and those who received placebo at 52 weeks [41, 46] (SMD 6.62, 95% CI -0.85 to 14.08, I^2^ = 0%) (Fig. 5).

### The remission of depression

**Fig. 6:**
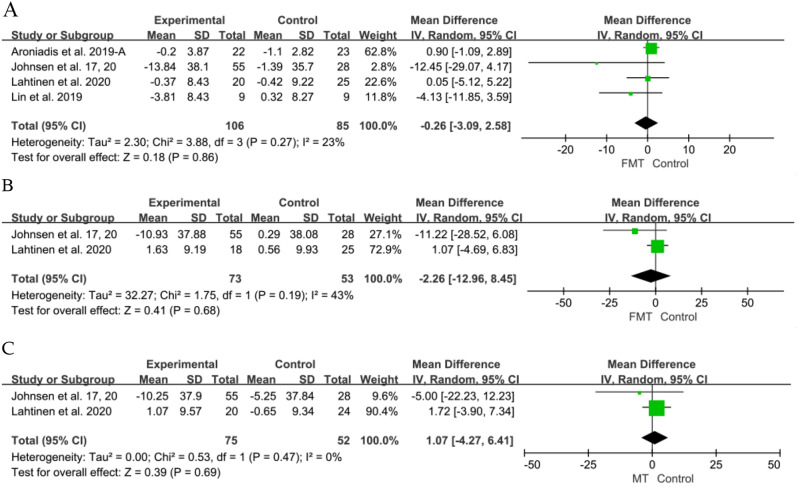
Forest plot of depression outcome between FMT and placebo. (**A**) 12 weeks (**B**) 24weeks (**C**) 54 weeks

The impact on depression was evaluated in four RCTs [6, 41, 46, 47]. Data from the four RCTs, comprising 106 participants in the FMT group and 85 in the placebo group, were extracted for analysis. Nonetheless, there was no significant difference observed between the FMT and placebo groups at the 12-week mark (SMD − 0.26, 95% CI -3.09 to 2.58, I^2^ = 23%), at 24 weeks from two RCTs [41, 46] (SMD − 2.26, 95% CI -12.96 to 8.45, I^2^ = 43%), and 54 weeks also from two RCTs(SMD 1.07, 95% CI -4.27 to 6.41, I^2^ = 0%) (Fig. 6).

### Adverse events

Seven out of ten RCTs provided data on total or individual adverse events (AEs). Aroniadis et al.‘s [6] study utilized a crossover trial design, where patients received both FMT and placebo capsules at different periods throughout the trial. To maintain consistency in the analysis, we excluded this study and pooled data from the other six [36, 40–44, 46] RCTs. No significant difference in the number of total AEs was found between the above two groups (*P* = 0.26). Individual adverse events were incompletely reported by individual RCTs.

The most commonly reported individual adverse events were constipation, diarrhea, nausea, abdominal pain/cramping/tenderness, and bloating. Constipation and abdominal pain/cramping/tenderness were found to be significantly higher in the FMT group compared to placebo(*P* = 0.0002, *P* = 0.0001 separately). No notable distinctions were detected in other prevalent individual adverse events (Table 3).

**Table 3:** The adverse events analyses comparing FMTwith placebo in IBS

	No. of RCTs	RR	95%CI	*P*	I^2^
Total AEs	6	2.36	0.53–10.61	0.26	88%
Diarrhea	6	3.72	0.77–17.97	0.1	70%
Nausea	6	1.25	0.72–2.18	0.43	0%
Bloating	4	1.24	0.40–3.82	0.71	25%
Constipation	4	7.81	2.68–22.80	0.0002	34%
Abdominal pain/cramping/tenderness	6	4.15	2.01–8.57	0.0001	38%

## Discussion

This systematic review and meta-analysis aimed to assess the effectiveness of FMT for the treatment of irritable bowel syndrome (IBS) and conducted subgroup analyses to identify factors influencing its efficacy. Our meta-analysis presents novel findings not previously reported : Firstly, we expanded upon the long-term results reported by El-Salhy et al. [36], encompassing all outcome measures, and additionally included the secondary outcome measures from Johnsen et al. [42]. Secondly, we examined the impact of FMT on depression, although there were no significant differences observed between the FMT and placebo groups.

In 2022, meta-analyses of randomized controlled trials (RCTs) examining the relationship between Irritable Bowel Syndrome (IBS) and Fecal Microbiota Transplantation (FMT) were published [33, 48]. Consistent with these meta-analyses, our findings indicate that FMT does not lead to a significant short-term and long-term global improvement in patients with IBS. However, we noted a significant improvement in global IBS symptoms with the administration of FMT via gastroscopy. Short-term observations proven a significant improvement in IBS-QoL between the FMT and placebo groups. However, no significant difference was noted between patients receiving donor FMT and those receiving placebo during long-term observations. Notably, the immediate effects of FMT have been observed on the initial day following administration [49]. However, a decline in the population of donor strains has been noticed 1.5-3 months post-FMT, resulting in a substantial decrease in the theoretical efficacy of FMT [1]. Hence, it may be necessary to undergo multiple FMT procedures. A study conducted by El-Salhy et al. demonstrated that patients who did not respond to a 30 g FMT showed notable enhancements in abdominal symptoms, fatigue, and quality of life (QoL) when they received a 60 g FMT after 3–4 months from the initial treatment. Moreover, Cui et al. [50] suggested a decrease in responsiveness over time following the FMT treatment period. Furthermore, in ulcerative colitis, FMT should be administered every three months [51]. Therefore, repeated and periodic FMT for IBS could significantly enhance and sustain its efficacy. While previous studies have suggested a suitable timeframe for repeated FMT of 3–4 months, further randomized controlled trials are necessary to establish the precise optimal duration for repetitive FMT.

In our subgroup analysis, we noted a significant improvement in global IBS symptoms among patients who underwent invasive FMT procedures, including nasojejunal tube, colonoscopy and gastroscopy. However, IBS patients who underwent FMT via oral capsules exhibited adverse effects. Despite an observed increase in microbial diversity in the FMT group, adverse effects were noted, studies by Aroniadis et al. and Halkjær et al. did not find any clinical beneficial effect on stool frequency, abdominal pain and stool form [52]. Furthermore, in their subsequent study, they identified sustained elevated levels of anaerobic bacteria, including Faecalibacterium, Prevotella, and Bacteroides [53], in the FMT group over the long term. However, the alterations in the microbiota triggered by oral FMT did not reach a level of significance to ameliorate IBS symptoms. Invasive FMT routes likely facilitate a greater delivery of donor stool dosage to the patients’ bowels compared to oral capsules, leading to improved abundance and diversity of the gut microbiome. The wider contact area provided by invasive procedures may facilitate the successful colonization of donor stool and the reconstruction of the gut microbiota.

In consideration of these findings, prioritizing invasive FMT routes over oral administration appears more advisable. The observed benefit of FMT for IBS patients in our meta-analysis, whether administered as a single or multiple doses, can be attributed to the use of colonoscopy or gastroscope as the delivery method in these studies.

Combining data from two randomized controlled trials (RCTs), FMT showed superiority over placebo in IBS patients who met the Rome IV criteria. However, no significant effect was observed when the criteria were based on Rome III. It should be noted that the division of Aroniadis et al.‘s crossover trial into two groups may have contributed to the observed superiority in the Rome IV criteria group, and thus, this result may not be entirely reliable.

Three of the RCTs focused on patients with IBS-M and IBS-D, while four of the RCTs included patients with IBS-D, IBS-C, and IBS-M. Additionally, two RCTs specifically included patients with IBS-D, and only one RCT included patients with IBS-D, IBS-C, IBS-M, and IBS-U. It is worth noting that three RCTs included different subsets of IBS patients.

IBS is known to significantly affect the quality of life, leading to decreased work productivity and increased healthcare utilization [54]. Our meta-analysis verify a potential improvement in the quality of life for individuals with IBS after 12 weeks of FMT. However, additional research is needed to comprehensively grasp the implications of this discovery. Concerning the long-term effectiveness of FMT, our analysis verified that FMT did not lead to a significant improvement in global symptoms at the 1-year follow-up. IBS is a chronic condition characterized by fluctuating and recurring symptoms over time [55], potentially accounting for the limited sustained impact of a single FMT on IBS. However, Holvoet et al. [45] demonstrated in their study that a second FMT was effective in 67% of IBS patients who initially responded to the first FMT. This indicates that repeated FMT could be considered as a viable long-term treatment option for IBS.

With the advancement of research on IBS, the understanding of the brain-gut axis has revealed a close connection between gut microbes and emotions. However, our analysis did not find a significant difference between the FMT and placebo groups at 12 weeks, based on data from four RCTs, nor at 24 weeks, based on data from two RCTs. Liu et al. observed similarities in fecal microbiota profiles between patients with depression and those with IBS-D [56]. However, our meta-analysis did not find any significant differences among studies that exclusively focused on patients with IBS-D. This suggests that further RCTs exploring the relationship between gut microbes and emotions are needed to obtain more conclusive results.

Regarding safety, the combined data indicated an elevated risk of diarrhea, constipation, and abdominal pain/cramping/tenderness after FMT compared to placebo. However, there were no notable distinctions in other prevalent individual adverse events such as nausea, bloating, and fatigue. A single serious adverse event was reported, involving a participant who experienced transient vertigo and nausea after FMT, requiring hospital observation. In recent reports, two patients who underwent FMT for indications other than IBS developed serious adverse events, one of which resulted in fatality [56]. These events have raised concerns about the safety of FMT for IBS, particularly considering that IBS is generally considered a benign gastrointestinal condition [57–59]. The individuals, aged 69 and 73, were immunosuppressed and had advanced liver cirrhosis and myelodysplastic syndrome, respectively. They received fecal capsules from a donor carrying an antibiotic-resistant strain of Escherichia coli [56]. It has been recommended to screen donors for extended-spectrum-beta-lactamase-producing E. coli and SARS-CoV-2 in feces to reduce the risk of known infections [59]. Furthermore, it has been suggested to restrict the selection of IBS patients for FMT to those without immune deficiencies, systemic diseases, severe illness, or ongoing immune-modulating medication to further minimize risks.

## Limitations

There are several limitations in our study. Firstly, all the chosen RCTs had a limited sample sizes, highlighting the need for larger studies to validate our findings. Secondly, significant heterogeneity was observed due to variations in donor selection, patient inclusion criteria, stool preparation, FMT administration routes, frequency, and doses among the RCTs. Therefore, standardizing the FMT experimental process is essential to minimize heterogeneity. Thirdly, important factors such as diet and concurrent medication use weren’t consistently analyzed or recorded across the studies, potentially influencing the outcomes. Moreover, the distinct inclusion criteria made it challenging to assess the effects of FMT on specific symptoms and different IBS subtypes. Consequently, more and larger standardized RCTs investigating FMT for the treatment of IBS are still warranted to address these limitations.

## Conclusions

In summary, our meta-analysis on FMT in IBS indicates a notable positive influence of FMT on short-term IBS-SSS and IBS-QoL. However, the long-term efficacy remains uncertain. The variations in clinical outcomes observed with FMT for IBS may potentially be linked to differences in the donor selection criteria, the route of administration, and the microbiome profile of donors.

### Electronic supplementary material

Below is the link to the electronic supplementary material.

Supplementary Material 1

## Data Availability

The original contributions presented in the study are included in the article/Supplementary Material, further inquiries can be directed to the corresponding author.
