# Supplementary material for: A meta-analysis of randomized controlled trials evaluating the effectiveness of fecal microbiota transplantation for patients with irritable bowel syndrome
Source: BMC Gastroenterol. 2024 Jul 5;24:217. doi: 10.1186/s12876-024-03311-x (PMC11225114; doi:10.1186/s12876-024-03311-x)
Supplement: Supplementary file 1 — Supplementary Material 1 [file 12876_2024_3311_MOESM1_ESM.docx]

| **Section and Topic** | **Item #** | **Checklist item** | **Location where item is reported** |
| --- | --- | --- | --- |
| **TITLE** | | |  |
| Title | 1 | 1 |  |
| **ABSTRACT** | | |  |
| Abstract | 2 | 1-2 |  |
| **INTRODUCTION** | | |  |
| Rationale | 3 | 3-4 |  |
| Objectives | 4 | 4 |  |
| **METHODS** | | |  |
| Eligibility criteria | 5 | 5 |  |
| Information sources | 6 | 4 |  |
| Search strategy | 7 | #1 Colonic Diseases[MeSH]  #2 IBS[Title/Abstract]  #3 ((irritable OR functional OR spastic) AND (bowel OR colon))[Title/Abstract]  #4 ((irritable OR functional OR spastic) AND (bloat* OR constipat* OR diarrh*))[Title/Abstract]  #5 #1 OR #2 OR #3 OR #4  #6 Fecal microbiota transplant*[Title/Abstract]  #7 Faecal microbiota transplant*[Title/Abstract]  #8 Fecal microbiome transplant*[Title/Abstract]  #9 Stool transplant*[Title/Abstract]  #10 FMT[Title/Abstract]  #11 Fecal transfusion*[Title/Abstract]  #12 Fecal bacteriotherap*[Title/Abstract]  #13 #6 OR #7 OR #8 OR #9 OR #10 OR #11 OR #12  #14 #5 AND #13 |  |
| Selection process | 8 | 6 |  |
| Data collection process | 9 | 6 |  |
| Data items | 10a | 5 |  |
|  | 10b | 5 |  |
| Study risk of bias assessment | 11 | 6 |  |
| Effect measures | 12 | 7 |  |
| Synthesis methods | 13 | 7 |  |
| Reporting bias assessment | 14 | 6 |  |
| Certainty assessment | 15 | 7 |  |
| **RESULTS** | | |  |
| Study selection | 16a | 9 |  |
|  | 16b | 8 |  |
| Study characteristics | 17 | 8 |  |
| Risk of bias in studies | 18 | 10 |  |
| Results of individual studies | 19 | 11-15 |  |
| Results of syntheses | 20 | 10-16 |  |
| Reporting biases | 21 | 10-16 |  |
| Certainty of evidence | 22 | / |  |
| **DISCUSSION** | | |  |
| Discussion | 23a | 17 |  |
|  | 23b | 20 |  |
|  | 23c | 21 |  |
| **OTHER INFORMATION** | | |  |
| Registration and protocol | 24a | Provide registration information for the review, including register name and registration number, or state that the review was not registered. |  |
|  | 24b | / |  |
|  | 24c | / |  |
| Support | 25 | 22 |  |
| Competing interests | 26 | 21-22 |  |
| Availability of data, code and other materials | 27 | / |  |

*From:*  Page MJ, McKenzie JE, Bossuyt PM, Boutron I, Hoffmann TC, Mulrow CD, et al. The PRISMA 2020 statement: an updated guideline for reporting systematic reviews. BMJ 2021;372:n71. doi: 10.1136/bmj.n71

For more information, visit: <http://www.prisma-statement.org/>
